# Barriers in participant recruitment of diverse ethnicities in the state of Kuwait

**DOI:** 10.1186/1475-9276-12-93

**Published:** 2013-11-20

**Authors:** Sufia Tariq, Catharine A Goddard, Naser Elkum

**Affiliations:** 1 Dasman Diabetes Institute, P.O. Box 1180, 15462 Kuwait City, Kuwait; 2 University of Dundee, Scotland, UK

## Abstract

**Background:**

High rejection rates of subject recruitments for research studies have been reported in immigrants in many countries. However, the barriers in recruiting members of the expatriate population in Kuwait have not yet been investigated. This study was therefore designed to identify barriers in recruiting expatriates for research studies in the state of Kuwait.

**Methods:**

A population-based cross-sectional study was conducted on expatriate subject’s aged 18 years and older living in Kuwait. Difference between groups of continuous independent variables was analyzed using the *t*-test. Different categories such as ethnicity and gender were compared using the chi-square test.

**Results:**

3460 (85.1%) participants were recruited and 617 (14.2%) refused to participate in the study while 2530 (38%) calls were unreachable from the total of 6607 calls placed. Younger subjects (mean age 41.1 years) were more hesitant to be part of the study compared to older participants. The rejections among South Asians was (41.8%), Arabs (32.6%), Southeast Asians (18.9%) while the others (6.6%) category was least to refuse among all the nationalities. Gender was not significantly associated with refusal.

**Conclusion:**

There is an acute lack of appropriate recording of the problems faced while recruiting the participants. The findings suggest important messages for the decision makers in the area of expatriate recruitments, to understand the challenge and design new strategies to overcome the problem of recruitment in the state of Kuwait for research studies.

## Introduction

Participant recruitment is considered to be one of the most difficult aspects of the research process [1]. Reaching target recruitment levels is necessary in research for both validity and generalizability to the target population [2]. Over the past 30 years it has been noticed that the number of subjects recruited into research studies is decreasing, this is especially true for epidemiological studies [3,4]. Recent estimates indicate that 85% of trials do not conclude on schedule due to low participant accrual, 60% to 80% of clinical trials in the United States do not meet their temporal endpoint because of challenges in recruitment, and 30% of trial sites fail to recruit even a single participant [1]. Overall the quality of descriptive studies regarding recruitment is poor, with little detail reported on who undertook recruitment [5].

With increasing numbers of people moving from one country to another, migrant’s health has become a key global public health issue. To address ethnic disparities in health status and care, it is important to include diverse ethnic populations in health research [6-8]. Several socio-cultural barriers that contribute to under-representation of expatriates in health research studies have been investigated [6-9]. Resources addressing these barriers in the literature are limited [8,10]. Failure to recruit sufficient participants from ethnic minorities in medical research studies is common, regrettable and highly inefficient.

The State of Kuwait has experienced rapid economic growth and socio-demographic and epidemiological transitions over the past six decades. This economic boom attracted many expatriates to come for work in Kuwait. According to the 2011 census, expatriates form 67% (2,514,107) of the population of Kuwait, with an average population growth rate of 6.7% annually. Despite the higher representation of expatriates in Kuwait, nationals constitute the majority of research participants and are predominant in prevention programs. As in other parts of the world the expatriate participant recruitment in health research studies is a big challenge similarly in Kuwait and to date we are not aware of any study that reports on recruitment issues among expatriate population in the state of Kuwait. The objectives of this study were therefore to describe, for the first time, the major barriers in recruitment and recommend strategies for successful recruitment of expatriates to health research studies in the state of Kuwait.

## Methods

### Setting

The Kuwait Diabetes Epidemiology Program (KDEP) was undertaken at Dasman Diabetes Institute (DDI) to estimate the magnitude of obesity, metabolic syndrome and Type 2 Diabetes among the expatriate population of Kuwait. This cross-sectional population-based survey was conducted with a random representative sample of adults (> 18 years) from multi-ethnic origin across the six governorates (strata) of the State of Kuwait. In this survey, we anticipated that the expected diabetic proportion would be 23% and would estimate this with a precision of 1.5%. Sample size was estimated as 3025 people. Taking into consideration 30% of non-response and rejection, a total sample of 3931 participants was planned to be randomly selected across the six strata with proportional allocation. For each stratum, a population frame included expatriates of both genders. A stratified random sampling technique was used for the selection of participants from the computerized register of the Public Authority of Civil Information (PACI). PACI is a government body which keeps all records of personal information of both Kuwaiti citizens as well as expatriates. The study was approved by the Scientific Advisory Council and Ethical Review Committee at DDI. This survey was carried out between June 2011 and August 2012.

### Calling

Enrolment was through a telephone call in which details of the study were explained to the potential participant and appointments for a study visit booked if the response was positive. Participants were informed about the importance of his/her participation as it would help scientists find new prevention and control strategies for obesity and related conditions which in turn would help in building a healthier community. Participants who agreed to join the study received free blood tests (fasting glucose, Hb1Ac, Liver function, Kidney function, Insulin level, Thyroid function, Vitamin D, CBC etc.), physical examination, and detailed medical report. Furthermore, the caller informed the participants that if any abnormalities were detected in their blood tests, free consultation would be provided by the in-house medical doctor and dietician. Different callers with different languages were hired and trained to handle calls with potential participants. There were four Arabic speaking callers, one Tagalog/English and one Urdu/Hindi speaking caller for placing the calls and booking the appointments. The phone numbers were distributed to callers according to their language expertise to ensure that the details of research could be explained to the potential participant as accurately as possible. An Applicant Interview Form (AIF) was given to the callers which they were required to complete accurately during their calls to the potential participants. The AIF included information regarding name, phone number, date of birth, gender, area of residence, nationality and language of preference. An appointment was booked for participants who indicated willingness to participate in the study during the phone conversation. Participants were then scheduled to visit DDI to meet a member of the research team, sign the consent form, be interviewed and have blood samples analyzed. A participant was considered recruited to the study if they attended their appointment at the DDI, signed the consent form and undertook the study.

### Codes assigned on each response

The KDEP team consisted of six callers, two supervisors, five interviewers and others. The callers assigned appropriate codes to each response after calling. To discover the factors affecting recruitment the study sample was taken from the KDEP caller’s log and the coded codes were recorded. Table 1 shows the codes assigned for each response. It was important for our research to study the AIFs completely so that we could get detailed information about age, ethnicity and gender of the potential participants who refused to participate.

**Table 1:** Codes assign and their explanation

**Codes**	**Explanations**
101	No answer
102	Busy ring tone
104	Answering machine
106	Switched off
107	Other problem
111	Participant will call back the caller on specific time
112	Participant will call back the caller at unspecified time
131	Language barrier
132	Needs tracing (person has moved to other place)
151	Final Refusal by participant during calling
171	Final no contact
172	Unlocatable
173	Unavailable (not present)
180	No such person
181	Final ineligible
183	No proxy available (too young)
191	Final other problem
204	Not home
211	Participant will call back the interviewer on specific time
212	Participant will call back the interviewer at unspecified
251	Final refusal by participant who failed to turned up after booking an appointment
281	Final ineligible
291	Final other problem

### Recruitment process and data collection

Calls were placed from the list obtained from PACI to book the appointments. The ‘no answers’ or ‘busy’ were called again at least five times on different days and timings to get hold of the person. Refusals from potential participants were coded and logged. The callers assigned code ′151′ as a refusal when they received direct refusal from the potential participant. The interviewer assigned code ′251′ when the participant dropped out in between the recruitment interview and the study or refused after booking an appointment when a reminder call was placed to them by interviewer. The criteria for assessing the recruitment reporting quality was adapted from Jadad [11]. The total number called were counted and how many among them were reachable or not were also calculated. The number and reason for dropouts and withdrawals was stated.

### Statistical analysis

The study participants were classified, based on age, ethnicity, and gender, recruited and refused. The demographic characteristics are presented against refusals using means ± SD or as numbers and percentages, as appropriate. Difference between groups of continuous independent variables i.e. age was analyzed using the *t*-test. Different categories like ethnicity and gender were compared using chi-square test. All statistical assessments were two sided and considered to be significant when *P*-value <0.05. The data analysis was carried out using SAS (version 9.2; SAS Institute, Cary, NC).

## Results

**Figure 1:**
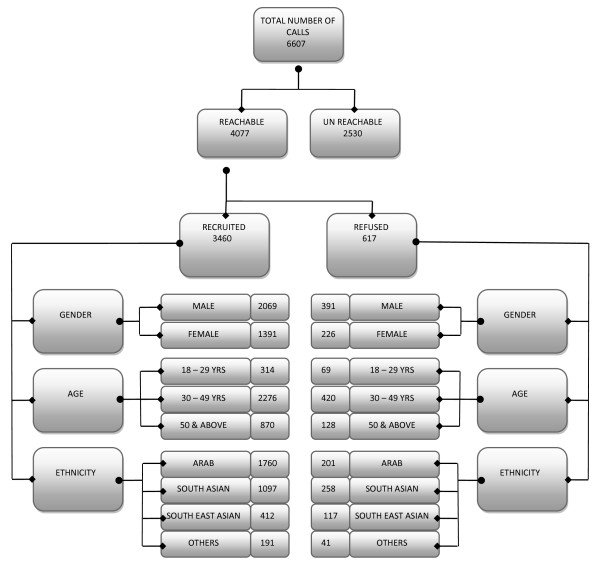
Recruitment process of KDEP.

A total number of 6607 calls were placed, from which 4077 (62%) were reachable and 2530 (38%) were unreachable. Among the reachable calls, 3460 (85.1%) were recruited and 617 (14.2%) refused to participate in the study. Figure 1 shows the recruitment process of KDEP. Most of the reasons for refusals given were related to time constraint (33.5%), not interested in the study (33.8%), restriction from employer (14.4%), personal reasons (9.3%), unavailability/travelling (6.2%) or health issues (2.7%). Table 2 illustrates the reasons given by the potential participants. We conducted further analysis on the refusal rates to find out how this is related to the gender, age and ethnicity of the potential participants and the reasons for refusals.

**Table 2:** Reasons of rejections

**Reasons of refusals**	**Rate (%)**
Do not have time to come for the study	33.46
Not interested in the study	33.85
Employer did not allow to have leave and come	14.40
Personal reasons	9.33
Going for vacations	6.23
Different reasons regarding health	2.72

### Age

**Figure 2:**
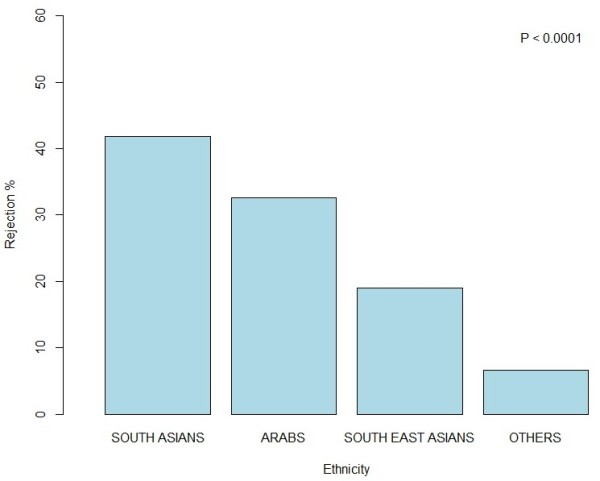
Rejection rate by ethnicity.

The overall mean age in our study was 42.5 years (SD = 10.2), the mean age for persons who refused was 41.1 years (SD = 10.3) while the mean age of those recruited was 42.8 years (SD = 10.1). Mean age of men was 42.9 ± 10.5 years and 41.9 ± 9.6 years for women. The youngest recruited person in the study was 18 years and the oldest recruited was 82 years of age. The rate of rejection decreased with age, and the highest proportion of rejection (47.9%) was detected in the below 40 year-old age group (Figure 2).

### Ethnicity

**Figure 3:**
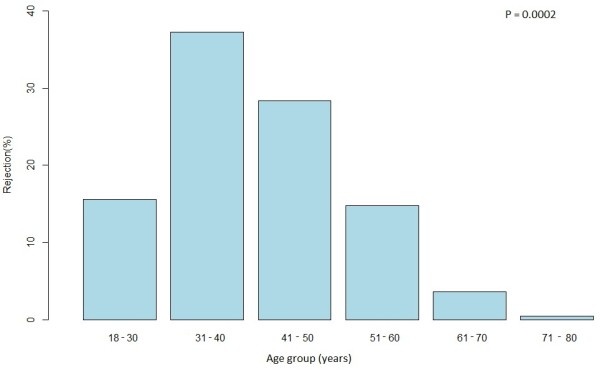
Rejection rate by age.

Kuwait is a multicultural country with many different nationalities (Arabs, Asians, Southeast Asians, Iranians, Europeans, and Americans etc.). As our target was to enroll various nationalities in the study, we therefore, categorized them into four ethnic groups i.e. Arabs, South Asians, South East Asians and Others (Iranians, Europeans, and Americans). Subjects originating from Arab countries such as Egypt, Syria, Lebanon, and/or Arab gulf countries (excluding native Kuwaiti nationals) were classified as Arabs. A total of 1961 (48.1%) Arabs were called from whom 201 (10.2%) refused to participate. Meanwhile, a total of 1355 (33.2%) of South Asians were called, from whom 258 (19.0%) refused, and among South East Asians a total of 529 (12.9%) were called, of whom 117 (22.1%) refused to participate. Lastly in the ‘Others’ category 232 (5.7%) participants were called of whom 41 (17.7%) refused. When the different ethnic groups were compared to each other there was a significant difference between the refusal rates with the highest number of refusals were from South Asians (41.8%) then Arabs (32.6%) after them South East Asians (18.9%) and the least refusals were seen from the Others categories (6.6%), *P* < 0.0001. Figure 3 shows the refusal pattern by ethnicity.

### Gender

2460 (60.3%) males were approached from whom 391 (15.9%) refused to participate due to different reasons. While among females 1617 (39.6%) were approached, 226 (13.9%) refused to participate *P* = 0.0946. This shows that gender of the potential participant did not play any significant role in recruitment or refusals.

## Discussion

Recruiting into epidemiological studies to ensure the sample population is representative of the age, gender and ethnicity of the target population remains a challenge. We sought to identify factors which prevent potential participants enrolling on studies within Kuwait. It is important that studies which seek to provide data to help the prevention and management of non communicable diseases, such as diabetes, which effect all ethnic communities within the country and increasingly younger age groups are able to include people from non–national ethnic backgrounds and young people groups which are traditionally most hard to recruit into studies.

Evidence with regard to study participation and age is not consistent [4]. Demaio provides evidence that older people are likely to refuse co operation on the first contact, but that refusals in the “age 50-years & above” category were somewhat more likely to be converted in follow-up attempts [12]. A more recent study by Dunn *et al.* shows that older persons are more likely to participate in the study [13]. Others reported higher participation rates among younger persons [14]. In the case of elderly persons, reluctance to participate in research may be caused by safety concerns and fears that they might become targets of financial swindles [2].

This study demonstrated a significant difference between the participation rates of different ethnic groups living in Kuwait. The ability to measure progress in ethnic disparities in health care is severely constrained by low level of participation of ethnic minority population in health related research [10]. Reasons for refusal to participate given by potential recruits included; living far away or busy at work and did not have enough time to come and participate. Perceived additional healthcare benefit is a reason often cited by participants for agreeing to take part in research. A benefit of participating in the KDEP study was receiving a full report following a blood test. However, in Kuwait the health care system provides blood tests free of charge or at very low cost so people can go to clinics or hospitals and receive the same care as part of their standard treatment. Therefore, our benefit of providing a blood test and report wasn’t sufficient incentive to participate. Distrust, culture, religiosity and economic group memberships are also considered factors which affect the willingness of members of different ethnic groups to participate in clinical trials [15].

Many studies reported clear evidence that women are more likely to participate in scientific studies than men [13,16]. Our study showed no difference in the rate of refusal between men and women but the reasons they gave were different. Reasons for refusal to participate given by potential recruits included: living far away, too busy at work, lack of interest as well as not having enough time to come and participate. Male potential participants reasons for non-participation were mostly that they cannot get leave or break from their work place. Whereas, many female potential participants, who refused, needed either their husbands’ consent or transportation to bring them to the research center. In addition the caller was not always able to talk to the intended potential recruit and other people declined on their behalf. Often the phone-number provided through PACI for an expatriate worker is that of the employer and in many cases the employer prevented us from talking to the person we wished to recruit, and refused on their behalf. In other cases the caller initially reached the spouse (husband/wife), father, mother, employer, sponsor of the potential participant and such persons who refused on behalf of the potential participant, mostly giving no reasons.

The main barrier faced during KDEP recruitment was that the PACI list was not updated. The PACI list usually consists of the name, civil identification number, governorate and phone numbers of the individuals. Although the PACI list was our centerpiece for sampling, we found that many records were either missing or incorrect. To best manage this issue, the callers were instructed to obtain up to date information from the person once the call was connected and if possible locate the correct number with the help of name and civil identification number so that representative sample could be achieved. Almost 2530 (38%) of called numbers were unreachable. Kuwait is a country where many expatriates do not have permanent residence, providing an explanation to why their number and address keeps changing. Language can play a crucial role as well. Most of the people called did not understand the research, until and unless a detailed description of study was explained in their local language. Language barrier may play a role in recruitment, as the study may not be fully explained and the potential participant is not able to understand the purpose of the study. The other major barrier faced was the mindset of the potential participants in Kuwait. People, generally are not aware or cognizant of the research culture and it is very important to increase the awareness about research and its benefits. Participation rates among cases in case–control studies are consistently higher than those among controls [17]. This was reflected in the KDEP study where almost 39.0% of participants with abnormal glycemic levels were recruited but only 3.1% of controls were recruited. The participant’s main reason for taking part in the study was to obtain long term personal benefits from our research institute as it has expertise and an exceptional reputation in diabetes, instead of the overall well-being of the community. Time constraints, lack of staff and training, lack of rewards and recognition, insufficiently interesting question are some of the other barriers in research studies [18]. Studies have also suggested that practitioner’s discussions about recruitment sometimes hide more fundamental doubts about the value placed on research and the real barriers to their participation [19]. Strategies creating a sense of collective ownership of projects between researchers and participants have been described as an important predictor of successful recruitment as long as 15 years ago [20].

### Recommendations

The problem for epidemiological research arises when participation is not randomly distributed across study groups [21]. Barriers to recruitment seem to differ among race and age subgroups, suggesting that recruitment strategies may need to be tailored to potential participants based upon demographic characteristics [2]. Different strategies can be adopted to encourage/facilitate full participation in research studies. The time required for participation is often a significant factor determining whether a person can participate in research. This is especially true for expatriates who are often in employment with long working hours and would suffer financial loss if they took time off work. The KDEP study required participants to come to Dasman Diabetes Institute (DDI) for an interview and a blood test. A strategy to enhance participation by reducing time required at the DDI would have been to conduct the interviews by phone so only the blood sample was taken at DDI. Alternatively the interview and sampling could occur at a range of places and a wider range of times to make it less time consuming for participants to attend by reducing travel times and providing more sessions.

When recruiting expatriate ethnic groups to a research study language is often an issue in preventing recruitment. To overcome this problem bilingual callers should be used, or an interpreter from the same nationality, to ensure the details of the research are conveyed accurately to the potential respondent. Sometimes it is possible to offer incentives without affecting ethical standards, for example transportation and a snack meal. Financial rewards above compensation for participant expenses should only be used with extreme caution. Ensuring you can contact the desired study participants is also important. Embassies are often a good source of information in addition to targeted publicity of the study and word of mouth. For this purpose social net-work sites and other sources such as brochures can be used in providing detailed information of the study beforehand. This approach might play a pivotal role in the recruitment of participants. Appropriate recording and reporting of the problems faced while recruiting and retaining the participants in research studies can help not only in understanding the challenge, but will also help in devising the strategies to overcome this problem [20]. Senior researchers are advised to evaluate every research project carefully and to use the outcomes regarding recruitment problems in future projects.

## Conclusion

Despite the low and inconsistent understanding of research and weak public support we were able to recruit almost 51% of the potential participants called, representing various age groups, ethnicities and genders. The adoption of standardized reporting guidelines for observational epidemiologic studies could improve the current practice of reporting epidemiologic research and could ultimately stimulate improvement in the methods of recruiting study participants and the research itself [22]. Whatever methods are used to contact potential study participants, the scientific need to achieve high response rates must be weighed against the ethical responsibility to respect individual privacy [2]. There is more work to be done in this part of the world to understand the limitations and barriers in recruitment of research participants in research studies.

## Competing interests

Nothing to declare, the authors declare that they have no conflicts of interest.

## Authors’ contributions

NE principal investigator handled data management, data analysis and interpretation, and wrote the manuscript. ST conceived the study and wrote the manuscript; CG data interpretation and critically revised the manuscript. All authors have read and approved the final version of the manuscript.
